# Maillard Reaction-Derived S-Doped Carbon Dots Promotes Downregulation of PPARγ, C/EBPα, and SREBP-1 Genes In-Vitro

**DOI:** 10.3390/molecules29092008

**Published:** 2024-04-26

**Authors:** Hanaa Hisham Habelreeh, Jegan Athinarayanan, Vaiyapuri Subbarayan Periasamy, Ali A. Alshatwi

**Affiliations:** Nanobiotechnology and Molecular Biology Research Laboratory, Department of Food Science and Nutrition, College of Food Science and Agriculture, King Saud University, P.O. Box 2460, Riyadh 11451, Saudi Arabia; 439204263@student.ksu.edu.sa (H.H.H.); jegan@ksu.edu.sa (J.A.);

**Keywords:** carbon dots, Maillard reaction, antioxidant, biocompatible, PPARγ, C/EBPα, SREBP-1, HMGCR, cytotoxicity

## Abstract

Carbon nanodots (CDs) are commonly found in food products and have attracted significant attention from food scientists. There is a high probability of CD exposure in humans, but its impacts on health are unclear. Therefore, health effects associated with CD consumption should be investigated. In this study, we attempted to create a model system of the Maillard reaction between cystine and glucose using a simple cooking approach. The CDs (CG-CDs) were isolated from cystine-glucose-based Maillard reaction products and characterized using fluorescence spectroscopy, X-ray diffractometer (XRD), and transmission electron microscope (TEM). Furthermore, human mesenchymal stem cells (hMCs) were used as a model to unravel the CDs’ cytotoxic properties. The physiochemical assessment revealed that CG-CDs emit excitation-dependent fluorescence and possess a circular shape with sizes ranging from 2 to 13 nm. CG-CDs are predominantly composed of carbon, oxygen, and sulfur. The results of the cytotoxicity evaluation indicate good biocompatibility, where no severe toxicity was observed in hMCs up to 400 μg/mL. The DPPH assay demonstrated that CDs exert potent antioxidant abilities. The qPCR analysis revealed that CDs promote the downregulation of the key regulatory genes, PPARγ, C/EBPα, SREBP-1, and HMGCR, coupled with the upregulation of anti-inflammatory genes. Our findings suggested that, along with their excellent biocompatibility, CG-CDs may offer positive health outcomes by modulating critical genes involved in lipogenesis, homeostasis, and obesity pathogenesis.

## 1. Introduction

Carbon dots (CDs), which were discovered by Xu et al. in 2004, are fluorescent, zero-dimensional nanomaterials with a size below 20 nanometers [1]. The CDs’ generation in food was first established in 2012 when Sk et al. demonstrated the presence of CDs in food caramels [2]. Since then, evidence has continued to emerge, and while some limitations remain, it has become clear that CDs are common in most daily foods, including meats [3,4,5,6], baked foods [7,8,9], beverages [10,11], and food dressings [12,13]. Thus, the daily and total consumption of CDs among the population is considerably high [14]. However, the health consequences of consuming food-formed CDs have not been sufficiently investigated.

Since their discovery, CDs have been considered a safe food component [2], and no severe toxic impacts of food-formed CDs have been observed in in vitro or in vivo models [4,6,9]. However, few in-vitro studies reported that when administered at high doses, food-formed CDs could impose cellular toxicity [7,8]. Indeed, CDs’ interaction with the biological system is facilitated by their unique characteristics, such as their small size, large surface area, and high surface-free energy. These characteristics create highly reactive surfaces that allow easy binding to functional molecules through chemical bonds. CDs were found to interact easily with biomolecules by forming hydrogen bonds. For instance, an in vivo study showed that CDs were rapidly absorbed by the intestine and interacted with human serum albumin (HSA) in the blood, changing its structural content [15]. High doses of baked lamb-derived CDs disturb energy metabolism in vitro [3].

While most studies have mainly focused on investigating the adverse effects of food-formed CD exposure, a growing body of literature demonstrates the excellent biocompatibility and low toxicity of CDs [16]. Thus, it is encouraging to change the research direction toward the potential of CDs to impose a positive health outcome. This study is one of the first attempts to shed light on the bright side of the presence of CDs in the daily consumed food.

Over the last few decades, great attention has been given to several molecular drug targets that can potentially prevent or overcome metabolic disorders. Specifically, nuclear receptors have attracted much attention due to their regulatory role in glucose homeostasis and lipogenesis [17]. Food-formed CDs showed an interesting capability in modulating gene expression in vitro [5,10], and therefore, the use of CDs in targeting nuclear receptors is worth studying.

Evidence suggests that the generation of CDs in food is highly related to Maillard reaction (MR) [18]. Thus, in this study, we synthesized CDs from the Maillard reaction between cysteine and glucose using simple boiling cooking techniques. The physicochemical characteristics of the produced CDs were systematically investigated, including their morphological features, composition, structure, and fluorescence behavior. Additionally, we have investigated the antioxidant activity and the cytotoxicity of CDs on hMCs. Lastly, we examined the modulation of CDs on the mRNA level of selected key metabolism genes involved in lipogenesis and obesity pathogenesis.

## 2. Result and Discussion

### 2.1. Synthesis and Physiochemical Characteristics

**Figure 1:**
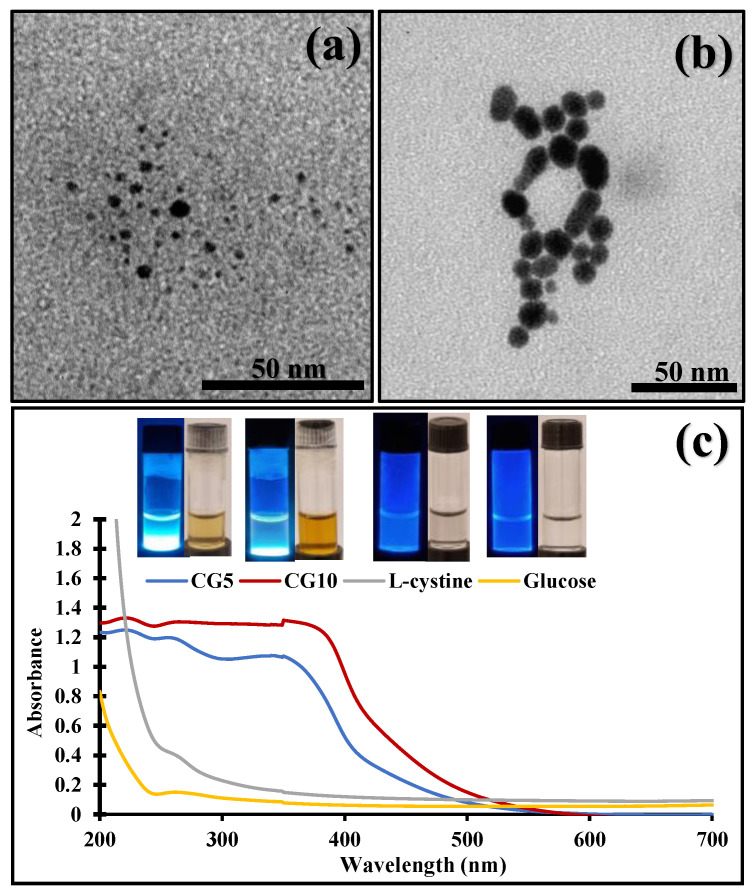
TEM images of (**a**) CG5, (**b**) CG10, and (**c**) UV-visible absorption spectrum of CG5 and CG10.

With a focus on simulating cooking methods, we designed a Maillard reaction (MR) model system to synthesize CDs. MR mainly occurs between amino acids and reducing sugars of food products during cooking. Glucose and cystine were selected precursors for MR due to their abundance in food material. Basically, the occurrence of a Maillard reaction is indicated by the development of brown color [19], and as the reaction time increases, the browning intensity of the product is increased [20]. Reaction time has been acknowledged as one influential variable in determining CDs’ physicochemical parameters. Thus, in this study, cystine–glucose CDs (CG-CDs) were synthesized in two different browning intensities achieved by 5 (CG_5_) and 10 min (CG_10_) of boiling. Most studies in CD synthesis have used prolonged thermal treatment to produce fluorescent CDs. For instance, the formation of CDs from glucose was only successful after 6 h of hydrothermal treatment [21]. Similarly, 10 h of hydrothermal treatment was used to fabricate MR-CDs from lysine and glucose. Also, studies on food-born MR-CDs reported the presence of CDs after 30 min of baking at various temperatures [8,22]. In this study, we confirm the generation of carbon dots at an early stage of MR where 5 and 10 min of boiling an aqueous solution containing MR precursors was sufficient to produce nanoparticles of less than 10 nm in size. This result is evident by TEM images, which show well-dispersed CDs with a size range of 2–13 nm (Figure 1).

As illustrated in (Figure 1c), CG-CDs exhibit strong blue fluorescence when exposed to UV light. These results confirmed the presence of CDs in the prepared samples. The UV–Vis absorption spectra of synthesized CDs exhibit the absorption bands at 226 and 263 nm, ascribed to π→π* transition of C=C and n→π* transition of C=O, respectively.

**Figure 2:**
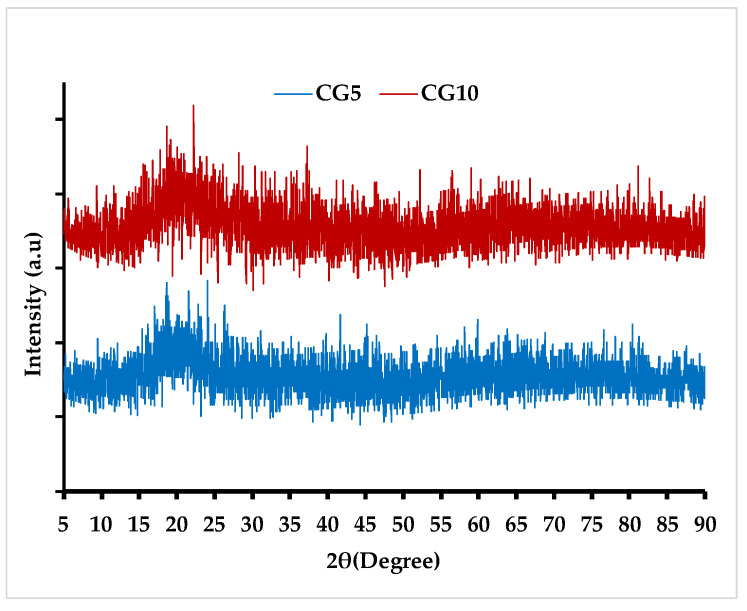
XRD pattern of Maillard-derived CDs.

The crystalline nature of CG-CDs was assessed using an X-ray diffractometer (XRD). The CG-CDs showed a broad peak at about 20°, corresponding to (002) of graphitic structure and indicating an amorphous nature of the synthesized CG-CD (Figure 2). The results of CDs studies [13,23] are in line with the current report.

**Figure 3:**
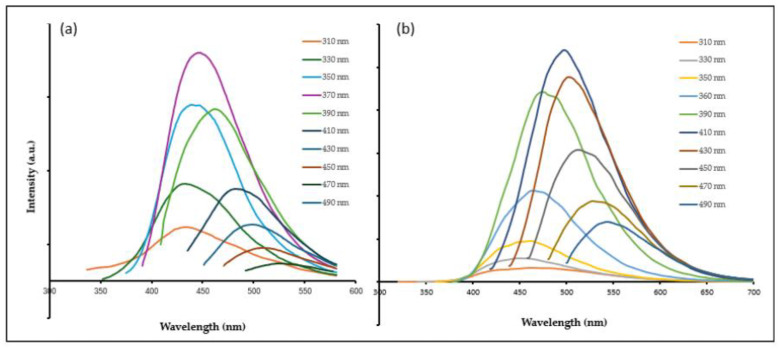
Fluorescence spectra of Maillard-derived CDs. (**a**) CG5 fluorescence spectra and (**b**) CG10 fluorescence spectra.

The fluorescence spectra (Figure 3) for CG5 and CG10 exhibited a bathochromic shift with a maximum emission wavelength of 450 and 490 nm at the excitation wavelength of 370 and 410 nm, respectively. The excitation-dependent photoluminescence behavior of CG-CDs observed in this study has been repeatedly reported in fluorescent carbon-based nanomaterials, which is attributed to differently sized CDs and multiple surface emissive trap statues [24].

**Figure 4:**
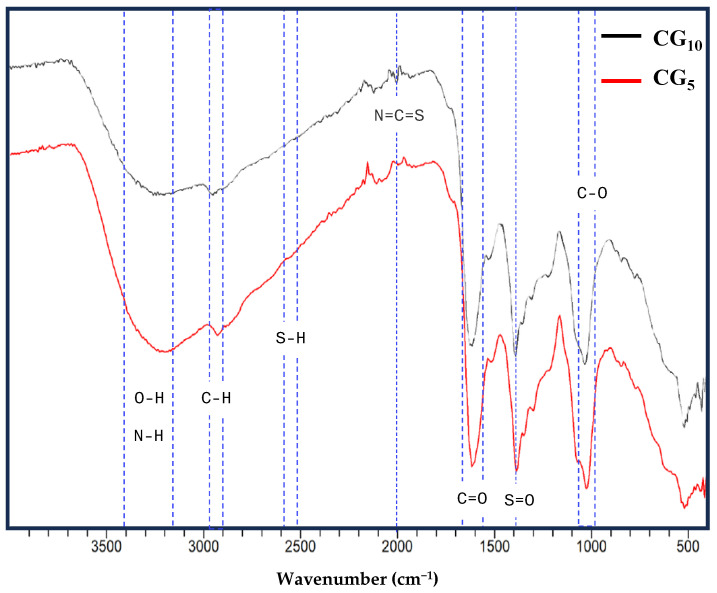
FTIR spectra of Maillard-derived CDs.

The elemental analysis indicates that CG-CDs are composed of C, O, and S. These results clearly indicate the presence of S-doping on CDs. FTIR analysis was performed to acquire clear information about the surface functional groups of CG-CDs. The FTIR spectrum of the CG_5_ and CG_10_ are shown in Figure 4. The broad band at 3400 cm^−1^ could be attributed to the stretching vibrations of the OH groups. The peak that appears between 2840 and 3000 cm^−1^ may represent C-H stretching. The weak peak between 2550 and 2600 cm^−1^ classically corresponds to the S-H group. Interestingly, this characteristic peak of S–H (thiol group) was not observed in the CG_10_ spectrum, which indicates that the S–H bond was cleaved and that a new N=C=S bond at 2000 cm^−1^ was formed. This peak appears stronger in CG_10_ compared to CG_5_. Bands shown at 1637 cm^−1^ and 1560 cm^−1^ were assigned to the presence of C=O and NH groups, which could be specific markers for the formation of primary amide and secondary amide, respectively. Amides are known to be major components of MR products [25]. Other bands at 1388 cm^−1^ and 1075 cm^−1^ may be attributed to S=O stretching and C-O stretching, respectively. Overall, the surface chemistry of CG-CD analysis indicates the presence of hydroxyl, amino, and carboxyl groups on the surface of MR-CDs, which explains their high-water solubility. Indeed, highly water-soluble CDs could readily pass through the intestinal wall. They can also link and facilitate the attachment of biomolecules and serve as nanocarriers. It should be noted that the impact of reaction time on the surface characteristic of CG-CDs is only noticed in the presence/absence of the thiol group.

### 2.2. Radical Scavenging Ability

**Figure 5:**
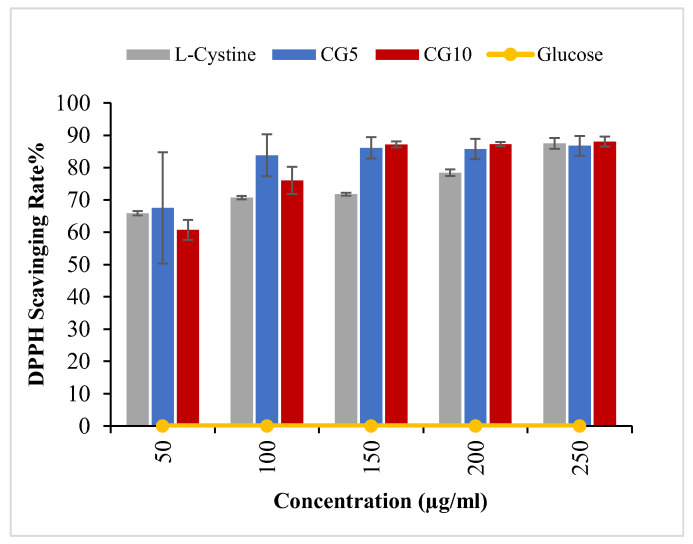
DPPH scavenging rate at different concentrations of CG-CDs in comparison to their precursors.

Radical scavenging activity of CG-CDs was analyzed using DPPH assay (Figure 5). The result revealed that CG_5_ and CG_10_ exhibited high scavenging ability against a DPPH of 70% and 60%, respectively, at low concentrations. Increased concentration achieved an even higher scavenging rate for both CG-CDs (up to 90%), reaching a plateau after that concentration, where further increases were not reflected in increased SR. Whereas studies on MRPs found that the increase in antioxidant activity of MR-derived products is positively related to browning intensity [20,21], we observed that CG_5_, synthesized with shorter heating time and a lower browning intensity, was a stronger antioxidant. When compared to CG_10_, this slight increase in SR could be attributed to the presence of a thiol group in the CG_5_ surface. This observation may indicate that the contribution of the thiol group to the overall antioxidant activity of CG-CDs is much weaker than that of the carboxyl group; the presence of a carboxyl group was considered a determining factor for the antioxidant activity of the MRPs derived from cystines [26]. MR-CDs reduce DPPH radicals by donating hydrogen to form a stable DPPH-H molecule [27]. The potency of the antioxidant activity of CG-CDs could be measured and compared with other antioxidants by determining the IC_50_, which represents the concentration at which a substance exerts half of its maximal inhibitory/scavenging effect. MR-derived nanoparticles synthesized by glucan–lysine [28] achieved a maximum IC50 of 128.81 μg/mL, whereas the IC50 of MR-CDs formed by glucose and Lys was 570 μg/mL [18]. CG-CDs in the current study achieved an IC50 of 32 ug/mL, indicating a highly strong antioxidant activity.

### 2.3. Cytotoxicity Assessment

**Figure 6:**
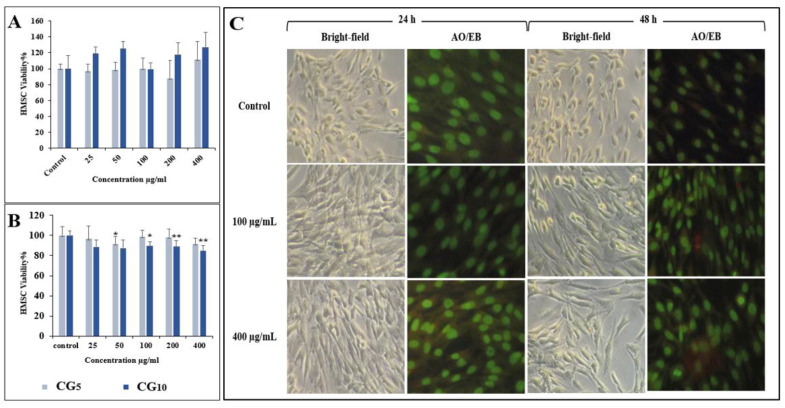
(**A**) Cell viability results of CG-CDs on the hMCs after 24 h and (**B**) 48 h. (**C**) Bright-field and AO/EB images of the hMCs were treated with CG10 for 24 and 48 h. * *p*-value > 0.05, ** *p*-value < 0.001.

**Figure 7:**
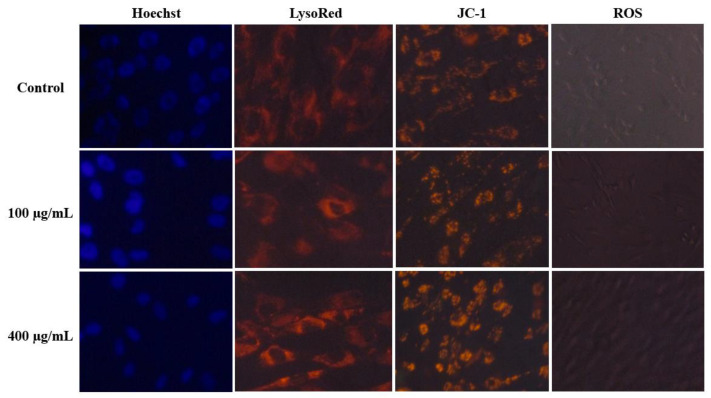
Hoechst, Lysored, JC-1, and ROS images of the hMCs treated with CG-CDs for 24 h demonstrating the biocompatibility and the harmless effect of CG-CDs on cellular morphology, lysosomal activity, mitochondrial membrane potential, and reactive oxygen species (ROS) generation.

Given that the CG-CDs were produced in a short cooking time, it is highly probable that these S-doped CDs are present in daily human food. Therefore, evaluating the cytotoxicity of the S-doped CG-CDs is highly important. Our in vitro cytotoxicity assessment revealed no significant changes on hMCs’ viability after 24 h of exposure to CG-CDs (Figure 6). However, with 48 h exposure, hMCs showed a reduction in cell viability by 20%, which was significant, although negligible, compared to the control (Figure 6). The cell viability results indicate that S-doped CDs do not adversely affect hMCs. The interaction between S-doped CDs and hMCs was studied using microscopic analysis. The bright-field microscopic analysis and AO/EB staining results are shown in Figure 6. The S-doped CDs treated and untreated cell morphological architecture are similar. Also, the AO/EB staining images show green and intact nuclei in S-doped CDs treated cells. These results are consistent with cell viability results. We also investigated the effect of CG-CDs’ exposure on lysosomes and mitochondrial membrane potential using LysoRed and Jc-1 staining, respectively (Figure 7). The result showed a healthy cell appearance and no loss of mitochondrial membrane potential.

ROSs are cellular metabolic coproducts that are normally generated in living cells. Under stress, the overgeneration of ROS molecules, followed by a sequence of feedback mechanisms, induces oxidative stress. Thus, we assessed the intracellular ROS level in hMCs after exposure to CG-CDs (Figure 7). The result showed that CG-CD exposure does not influence intracellular ROS basal levels compared with the control. Overall, the current in-vitro assessment did not detect any evidence of cytotoxicity associated with exposure to CG-CDs derived from MR. These results are consistent with other research that found that CDs extracted from grilled fish have excellent compatibility when assessed in MC3T3-E1 cells [6].

Reaction time seems to be an influential factor in determining the extent to which MR-CDs can impose toxic effects at high doses. While CG10 imposed a slight decrease in cell viability, although negligible, CG5 had no impact at all. The overall result of our cytotoxic assessment revealed that no serious toxicity was observed on hMCs when exposed to CG-CDs up to 400 μg/mL. The study’s results are in line with previous studies, where food-formed CDs have a low toxicity profile [2,4,5] unless administered at extremely high doses [7,15].

### 2.4. Gene Expression

**Figure 8:**
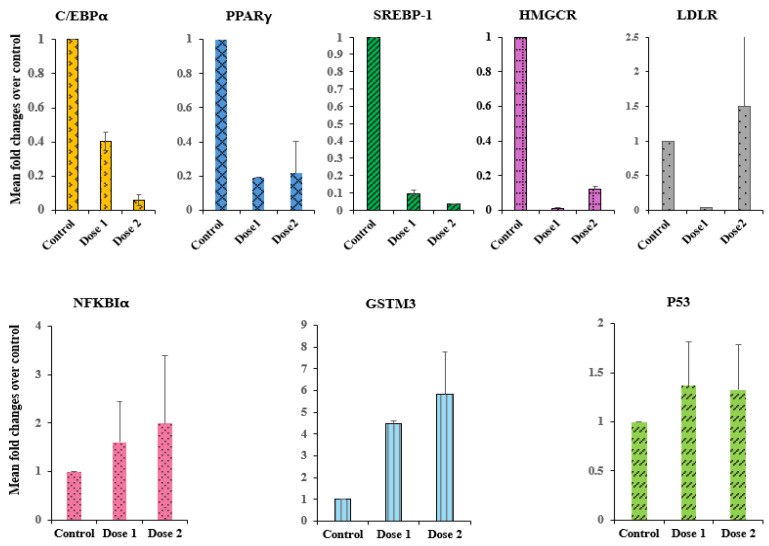
Modulation of mRNA transcription level of C/EBPα, PPARγ, SREBP-1, HMGCR, LDLR, NFKBIα, GSTM3, and P53 genes by CG_10_. Dose1 (100 μg/mL), Dose2 (400 μg/mL).

The potential effect of food-formed CDs on lipogenesis and obesity-related genes has not been previously studied. Sterol regulatory element-binding protein-1 (SREBP-1) is a crucial transcription factor in regulating genes involved in the de novo lipogenesis and glycolysis pathways. SREBP-1 level significantly increases in obese patients and animal models of obesity and type 2 diabetes [29]. In fact, a large number of reports have implicated the SREBP transcription factor in the pathogenesis of metabolic disorders [30]. Also, SREBPs regulate the rate-limiting lipogenic and cholesterogenic genes, such as HMG-CoA reductase and the LDL receptor [30]. This study investigated the effect of CG-CDs on the transcriptional level of SREBP-1 and its downstream genes LDLR and HMGCR. CG-CDs significantly attenuated the expression of SREBP-1 gene and reduced transcription levels of the genes encoding HMG-CoA synthase (Figure 8). Although a previous in vitro study reported that CDs induced an increase in the mRNA of SREBP-1 and its target genes, this increase was ROS related and it is brought about by relatively high-dose treatment that induced cellular toxicity [3]. ROS accumulation leads to SREBP activation in vitro [31]. According to pharmacological studies, a vast number of studies in human and animal models has strongly proposed that upregulation of SREBPs has a central role in the pathogenesis of the metabolic syndrome, and therefore, compounds that are capable of redeciding the activity of SREBPs may be useful for the treatment of this metabolic syndrome and may improve its related complications [32].

In addition, SREPB-1, Peroxisome Proliferator-Activated Receptor Gamma (PPARγ), and CCAAT-Enhancer Binding Proteins (C/EBPα) are critical transcription factors in adipogenesis [33]. Adipogenesis plays a vital role in the development of obesity and metabolic disorders [34]. Our results showed that exposure to CG-CDs reduced the expression of the adipogenic transcription genes PPARγ and C/EBPα by 81% and 94%, respectively. Inhibition of PPARγ in ob/ob mice prevents weight gain and adipose tissue deposition in mice and reduces hyperglycemia and hyperinsulinemia [35,36]. Cell-surface mediated receptor interactions between CG-CDs and cell surface receptors like G-protein coupled receptors (GPCRs), receptor tyrosine kinases (RTKs), or cytokine receptors may initiate intracellular signalling cascades that modulate the activity of transcription factors regulating genes involved in lipid metabolism, leading to the downregulation of key genes such as C/EBPα, PPARγ, SREBP-1, and HMGCR.

In addition, the transcription level of NFKB Inhibitor alpha and P53 genes was enhanced with exposure to CG-CDs, which probably indicates a potential anti-inflammatory activity. Also, CG-CDs elevate the expression of GSTM3 by six-fold. The GSTM3 gene, encoding glutathione S-transferase, is involved in the detoxification of electrophilic compounds, including carcinogens, therapeutic drugs, environmental toxins, as well as oxidative stress products, by combination with glutathione [37]. The upregulation of GSTM3 may further support the former antioxidant assessment and strengthen the evidence that CG-CDs exhibit antioxidant properties in vitro.

Taken together, the result of the current study suggests that CG-CDs exert antioxidant and anti-inflammatory activity and could potentially act on the molecular level as a preventive agent against obesity and its related metabolic disorders. Moreover, CG-CDs may offer a cholesterol-reducing effect by downregulating HMG-CoA reductase and, therefore, inhibiting the endogenous synthesis of cholesterol. This is the first study reporting a potential anti-cholestrogenic, anti-obesity effect of MR-CDs attributed to direct modulation of lipogenesis and adipogenesis gene expression.

While additional supporting studies are needed to confirm the capability of CG-CDs against adiposity and cholesterol biosynthesis, this study offers valuable insight that the biocompatible and food-originated CG-CDs with potent antioxidant activity could be a promising candidate for future research as a potential therapeutic agent against obesity and metabolic disorders. Future in-vivo studies are required to examine the effect of CG-CDs on lipogenesis, cholesterol de novo synthesis, and obesity biomarkers.

## 3. Materials and Methods

### 3.1. Materials

l-cystine was purchased from Alpha Chemika, Mumbai, India. Alpha-d glucose was obtained from Acros Organics, Fair Lawn, NJ, USA. Human mesenchymal stem cells (hMCs) were obtained from the American Type Culture Collection (ATCC, Manassas, VA, USA). MTT dye [3-(4,5-dimethylthiazol-2-yl)-2,5-diphenyltetrazolium bromide], acridine orange (AO), ethidium bromide (EB), and fetal bovine serum (FBS), and DMEM were obtained from Sigma, USA. Trypsin and other cell culture materials were obtained from Gibco, Paisley, UK. cDNA kit and primers were purchased from QIAGEN, Hilden, Germany.

### 3.2. Synthesis of CDs via Maillard Reaction

Around 2 g of cystine and 1 g of glucose were added to 30 mL of distilled water. Then, the aqueous mixture was allowed to boil until the browning reaction was visualized and the time was noted. Two browning intensities were acquired after 5 and 10 min of boiling. The resulting fractions were re-dissolved in 50 mL of distilled water. To eliminate insoluble materials, the obtained Maillard products were centrifugated at 10,000 rpm for 10 min. Next, the supernatants were filtered using a syringe filter (0.22 μM). Subsequently, the acquired filtrates were dialyzed against distilled water with a membrane with the molecular weight cut off at 3500 Da for purification. The aqueous solution containing CDs was then freeze-dried for further characterization.

### 3.3. Characterization of CDs

The CDs’ crystalline structure was analyzed using a powder X-ray diffractometer (XRD). The data were captured in the 2 θ range of 5–60° using a Rigaku D/max-2550 diffractometer equipped with Cu Kα radiation (λ = 1.5418 Å). The structural features of CDs were observed employing transmission electron microscopy (TEM) (JEM-1400 Plus, JEOL, Japan). The fluorescence property of CDs was assessed with different excitations using a fluorescence spectrophotometer (Cary Eclipse fluorescence spectrophotometer, Santa Clara, CA, USA). We examined the functional groups on CDs’ surfaces using FTIR spectroscopy (Nicolet 6700 infrared spectroscopy) from 4000 to 400 cm^−1^ [38].

### 3.4. Free Radical Scavenging Activity

The antioxidant activity of CDs was measured using a DPPH assay. In a 96-well microplate, 150 μL of DPPH solution (0.2 mM in absolute ethanol) was added to 50 μL of CG-CDs solution of various concentrations (50, 100, 200, and 250 μg/mL). After incubation in the dark for 30 m, the absorbance was measured at 517 nm. The following equation calculated the inhibition ratio (%):

$$Inhibition\;ratio\%=\left(OD\;of\;control-OD\;of\;sample\right)\times 100÷OD\;of\;control$$

where the control is the DPPH solution added to the ethanol, and the sample is the DPPH solution added to the CG-CDs or the ascorbic acid solution (as a positive control).

### 3.5. Cell Culture

The human mesenchymal stem cells (hMCs) were cultured in Dulbecco’s modified Eagle medium (DMEM) supplemented with 10% (*v*/*v*) fetal bovine and 1% penicillin/streptomycin serum using T75 flasks. The flasks were incubated in a CO_2_ incubator at 37 °C with 5% carbon dioxide.

### 3.6. Cell Viability Assay

In a 96-well plate, hMCs cells were seeded in 200 μL of medium at a density of 15,000 cells per well. Next, hMCs were exposed to CDs at different concentrations (0, 25, 50, 100, 200, and 400 μg/mL) and incubated for 24 and 48 h. At the end of incubation time, 20 μL of MTT solution was added to each well and incubated for 6 h at 37 °C. Then, the media were discarded, and 100 μL of DMSO was added to each well. The absorbance was monitored at 570 nm (measurement) and 630 nm (reference) using a 96-well plate reader (Bio-Rad, Hercules, CA, USA). The experiment was carried out in triplicates to calculate the mean. The percentage of cell survival rate was calculated using the formula:

$$Cell\;viability\%=\frac{Mean\;OD\;of\;treated\;cells}{Mean\;OD\;of\;untreated\;cells}\times 100$$

### 3.7. Acridine Orange/Ethidium Bromide Staining

The hMCs were exposed to different CD doses for 24 and 48 h. At the end of the incubation period, the media were carefully removed. Next, the cells were stained using dual-stain acridine orange/ethidium bromide (AO/EB). Stained cells were subsequently visualized and photographed under fluorescence microscopy.

### 3.8. Assessment of Mitochondrial Membrane Potential (MMP)

By using the fluorescent probe JC-1 and fluorescence microscopy, changes in mitochondrial membrane potential were investigated. hMCs were plated at a density of 50,000 cells per well in 12-well plates. Then, cells were exposed to CG-CDs (Control, 100, and 400 μg/mL) for 24 h. After incubation, the media was discarded, and JC-1 was added to each well to be incubated for 30 min. After staining, the MMP activity of hMCs was analyzed using fluorescent microscopy (Carl Zeiss, San Diego, CA, USA), where the emission of green fluorescence indicates a low MMP, and red-orange fluorescence emission indicates a high MMP.

### 3.9. LysoRed Staining

hMCs were exposed to CDs at 100 and 400 μg/mL doses and incubated for 24 h. Next, the cells were labelled with LysoRed and allowed for 15 min to be observed under a fluorescence microscope.

### 3.10. Hoechst Staining

The hMCs were exposed to different concentrations of CDs for 24 h. The media was removed, and Hoechst 33,342 stain application was added to every well. Then, the plate was kept at 37 °C for 15 min by 15 min incubation in the dark. Nuclear changes were observed using a fluorescence microscope.

### 3.11. ROS Assay

To explore the effect of CDs on intracellular ROS generation in hMCs, DCFDA (2′,7′-dichloro fluorescein diacetate) fluorescent probes were used according to the manufacturer’s instructions (ThermoFisher Scientific, Waltham, MA, USA). ROS causes dichlorofluorescein (DCF) to exhibit high fluorescence when oxidized. After 24 h of CD exposure, media were removed, and the DCFDA dye was applied. Next, the cells were incubated in the dark at 37 °C for 30 min to be examined under a fluorescence microscope.

### 3.12. Gene Expression Analysis

The most effective fraction of the produced CDs was selected gene expression analysis. hMCs cells were grown and treated with CDs for 24 h. After treatment, cDNA was synthesized with the Fastlane^®^ Cell cDNA kit (QIAGEN, Germany). The targeted genes’ mRNA levels (GSTM3, NFκBIA, P53, SREBP1, HMGCR, PPARγ and CEBPα) were assessed using GAPDH as a housekeeping gene. In each well of a 96-well PCR plate, 12.5 μL of the master mix, 2 μL of cDNA (500 ng), 2 μL of primer, and 4 μL of RNase-free water were mixed. The reaction mixture was run with real-time PCR for 40 cycles. The acquired data were analyzed using the comparative threshold (Ct) method. Target gene expression was measured in treated cells compared to the control cells. Then, the target gene expression level was measured and normalized to the expression of GAPDH, the reference gene. The expression ratio of the reference gene to the target genes, as well as the relative expression levels, was calculated using the following formulas:

$$∆Ct=\left(Target\;genes\right)-Ct\left(GAPDH\right)$$

$$∆∆Ct=∆Ct\left(treated\right)-∆Ct\left(control\right)$$

The resulting values were used to plot the expression of the targeted genes using the expression 2^−ΔΔCt^.

### 3.13. Statistical Analysis

Experiments were carried out in triplicate, and the obtained data are presented as mean ± standard deviation (SD). A paired t-test is used for statistical significance. A *p*-value of ≤0.05 was considered statistically significant. The data were calculated using Microsoft Office Excel software (Microsoft Excel® for Microsoft 365 MSO (Version 2403 Build 16.0.17425.20176) 64-bit).

## Data Availability

The data used to support the findings of this study are included within the article and will be available to the readership of this journal upon publication.
